# Multi Characteristic Analysis of Vascular Cambium Cells in *Populus euphratica* Reveals Its Anti-Aging Strategy

**DOI:** 10.3390/plants13243549

**Published:** 2024-12-19

**Authors:** Xiaoli Han, Zhongshuai Gai, Jianhao Sun, Juntuan Zhai, Chen Qiu, Zhihua Wu, Zhijun Li

**Affiliations:** 1 Xinjiang Production and Construction Corps Key Laboratory of Protection and Utilization of Biological Resources in Tarim Basin, Desert Poplar Research Center of Tarim University, College of Life Science and Technology, Tarim University, Alar 843300, China; lilyan0509@163.com (X.H.);; 2 College of Life Sciences, Zhejiang Normal University, Jinhua 321004, China

**Keywords:** *Populus euphratica*, senescence, vascular cambium, transcriptome

## Abstract

All multicellular organisms undergo senescence, but the continuous division of the vascular cambium in plants enables certain tree species to survive for hundreds or even thousands of years. Previous studies have focused on the development of the vascular cambium, but the mechanisms regulating age-related changes remain poorly understood. This study investigated age-related changes in the vascular cambium of *P. euphratica* trees aged 50 to 350 years. The number of cambium cells in the 50-year-old tree group was 10 ± 2, while the number of cambium cells in the 200-year-old and 350-year-old tree groups significantly decreased. The thickness of the cambium cells exhibited a similar trend. In addition, the net photosynthetic and transpiration rates continue to increase with age, but no notable differences were found in factors like average leaf area, palisade tissue thickness, and stomatal density. A total of 6491 differentially expressed genes (DEGs) were identified in the vascular cambium of *P. euphratica* at three distinct ages using RNA sequencing. The expression patterns of DEGs associated with cell division and differentiation, lignin biosynthesis, plant hormones, and transcription factors were analyzed. DEGs related to XTH, EXP, PAL, C4H, ABA, Br, GA, and others are highly expressed in older trees, whilst those encoding expansins, kinases, cyclins, 4CL, Auxin, Eth, SA, and others are more prevalent in younger trees. Gene family members, such as *NAC*, *MYB*, *HD-ZIP III*, *WRKY*, and *GRF*, have various regulatory functions in the vascular cambium. The findings offer insights into how ancient *P. euphratica* trees maintain vitality by balancing growth and aging, providing a foundation for future research on their longevity mechanisms.

## 1. Introduction

The aging of trees is a multifaceted process that involves a decline in their overall external appearance, internal structure, and physiological functions. It is a complex physiological phenomenon, with aging representing merely the final phase of a plant’s development [1]. Annual flowering plants show signs of aging and death after they bloom. By contrast, aging in long-lived perennial plants is challenging to notice unless they experience substantial environmental stress that causes them to age or die. Although trees exhibit considerable adaptability, their growth and longevity are restricted not only by biological and environmental pressures but also age-related structural limitations [2].

Studies on trees of various ages have revealed notable morphological differences between juvenile and adult specimens in certain species. One key distinction is observed in leaf shape; for instance, the young leaves of the woody plant *O. europaea* are smaller and rounder than the leaves of mature plants [3]. Research has shown that young *Eucalyptus globulus* leaves are oval with gradually pointed tips, lack petioles, and have a waxy cuticle layer. By contrast, mature leaves are lanceolate, possess petioles, and do not have a waxy cuticle [4]. *P. euphratica* is known for its heteroplasticity. Studies indicate that this species displays four distinct leaf shapes at various developmental stages or within different canopy structures: strip-shaped leaves (Li), lanceolate leaves (La), ovate leaves (Ov), and broad ovate leaves (Bo), which reflect age-related variations [5]. Similarly, tree age significantly affects tree body indicators (shoot length, shoot thickness, shoot height-to-diameter ratio, bark thickness, crown width, and breast height diameter) and leaf morphology and structure indicators (leaf length, leaf width, leaf shape index, petiole length, leaf area, and leaf thickness [6]. The research conducted by Li Zhijun et al. on the structural characteristics of the alien leaves of *P. euphratica* and their correlation with breast height diameter revealed that as *P. euphratica* plants mature, their leaf area, thickness, dry weight, and dry matter content progressively increase, whereas the specific leaf area steadily declines [7]. Alongside the aforementioned modifications, recent studies have examined how functional traits like the net photosynthetic rate of plants, hydraulic conductivity, and leaf water potential vary with the age of trees [8]. As trees age, the net photosynthetic rate often decreases, which may be due to factors such as decreased efficiency of the key enzyme Rubisco in photosynthesis, aging chloroplast structure, limited stomatal function, or insufficient nutrient supply [9]. Studies on coniferous tree species indicate that as trees age, the net photosynthetic rate of their leaves declines. However, findings regarding broad-leaved tree species are not consistent [10]. The reduction in transpiration rates in older trees hampers the upward movement of water and nutrient distribution, which, in turn, influence the photosynthetic efficiency of the leaves [11].

The vascular cambium is a type of meristematic tissue found between the xylem and phloem in the stems and roots of gymnosperms and dicot plants. This continuous ring of stem cells in the vascular cambium is responsible for the growth in the circumference of trees and the yearly renewal of their vascular tissue [12]. Older trees experience delayed woody development, have a shorter growing season, and exhibit a slower growth rate, leading to a reduced number of xylem cells. Previous studies on the vascular cambium of *Cunninghamia lanceolata* of different ages have shown that there are significant differences in the number and cell wall thickness of vascular cambium cells in 3-year-old, 13-year-old, and 35-year-old *C. lanceolata* with increasing tree age [13]. Subsequently, Wang et al. found that the activity of the vascular cambium in *Ginkgo biloba* at 20, 200, and 600 years old decreased with increasing tree age [14]. These results suggest that the activity of the cambium meristem in woody plants is age-dependent, making it a significant indicator of a tree’s growth vitality [15]. Earlier research has primarily concentrated on how vascular cambium activity is regulated by seasonal changes and the genetic framework that supports stem cell maintenance. By contrast, studies examining the relationship between vascular cambium and tree age are limited. Thickening the plant axis is a fundamental function of the vascular cambium, and the balance between cell division and differentiation is essential for achieving this function. This balance is dynamic and is regulated by environmental factors and internal rhythms. Monopteros (MP)/Auxin Response Factor 5 (*ARF5*) is a key regulatory factor in auxin signaling, playing a crucial role in the differentiation of precursor cells in the primitive layer. In MP mutant embryos, the primitive layer was absent, and the vascular bundles were reduced and discontinuous [16]. Cytokinin is a crucial regulatory factor in cambium development. The quadruple *Arabidopsis thaliana* mutant atipt1;3;5;7, in which four genes encoding cytokinin biosynthetic isopentenyl transferases are disrupted by T-DNA insertion, was unable to form cambium and exhibited reduced thickening of the root and stem [17]. Studies have shown that *COI1*, *MYC2*, *JAZ7*, and the touch-sensitive gene *JAZ10*, which are part of the JA signaling pathway, play a regulatory role in the formation layer. The beneficial impact of JA on cambium activity supports its role in promoting secondary growth and suggests that JA signaling initiates cell division in this specific context [18]. The homologous genes *PdBRI1-1, -2, -3*, and *-6* in *A. thaliana* were predominantly expressed in the cambium zone, the thin-walled tissue of primary xylem, and the developing cells of secondary xylem and phloem, as shown by situ hybridisation analysis. While blocking BR signaling may not affect the division of cambium during radial stem growth in Poplar, it change the differentiation of xylem and phloem tissues in the primary cambium cells of Poplar stems [19].

Certain transcription factors are involved in the differentiation of the vascular cambium. Recent studies have identified a regulatory pathway involving CLE41/44-TDR/PXY-WOX4 that helps sustain cambium activity and encourages cell proliferation within the vascular cambium [20]. *ATHB8*, which belongs to the *HD-ZIP III* gene family of transcription factors with leucine zipper homologous domains, is primarily found in the cambium and xylem. When *ATHB8* is overexpressed, it can enhance the process of lignification [21,22]. The development of xylem cells and the formation of cell walls are controlled by the *NAC* (*NAM, ATAF1/2, CUC1/2*) and *MYB* transcription factor families. Among them, the *NAC* transcription factor *SND1* is uniquely expressed in mesenchymal fibroblasts, and its activity is suppressed, which impedes the production of fibroblast cell walls [23]. *MYB46* and *MYB83* are genes that *SND1* targets, and they function downstream to enhance the production of secondary walls [24]. *MYB58*, *MYB63*, and *MYB85* are targets of *SND1* and are uniquely expressed in fibroblasts and ductal cells, where they play a role in promoting the expression of genes associated with lignin synthesis [25].

*P. euphratica* is the oldest and most primitive tree species in the Salicaceae family, with a history spanning more than 3 million years. It is known for its remarkable longevity, often described as ‘not dying for a thousand years, remaining upright for a thousand years after death, and not decaying for a thousand years after falling.” This tree possesses exceptional traits, including resistance to drought, cold, salt, alkali, and sand burial, which set it apart from typical tree species. A preliminary survey of the *P. euphratica* population conducted in the laboratory indicates that amongst approximately 670 ancient *P. euphratica* trees in Xinjiang, around 203 of them are over 500 years old. This study compared the morphological structure and photosynthetic physiological characteristics of *P. euphratica* at different ages to understand the relationship between tree age, morphology, and physiology. Additionally, the impact of aging on longevity was evaluated by examining changes in the vascular cambium characteristics of *P. euphratica* at both cellular and molecular levels. By measuring cambium activity and the transcriptional regulatory network based on tree age, differential genes in the vascular cambium of trees at different ages were identified, revealing how ancient *P. euphratica* maintains its growth ability by balancing the processes of growth and aging.

## 2. Results

### 2.1. Evaluation of the Growth of Poplar at Different Ages

**Figure 1:**
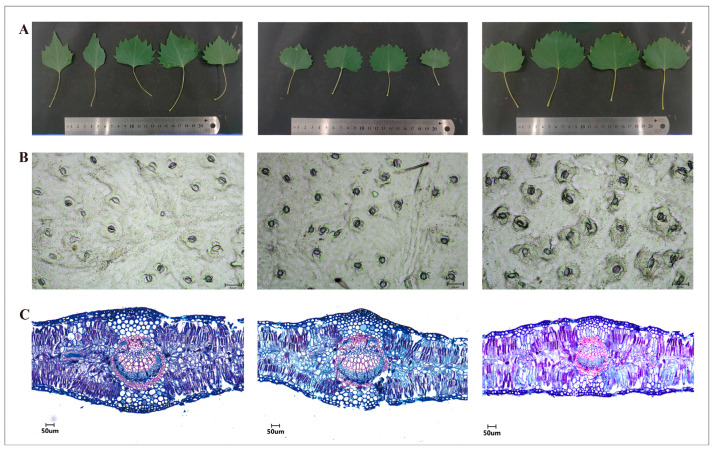
Phenotypic analysis of *P. euphratica* trees of different ages. (**A**) Leaf morphology of *P. euphratica* at different ages; (**B**) Stomatal density of *P. euphratica* leaves at different ages; (**C**) Anatomical structure of leaves of different tree ages. The tree ages from left to right are MY (50 years), MM (250 years), and MO (300 years).

The structure and functional traits of leaves have a direct impact on photosynthesis, influencing how plants acquire and use resources, which indicates their ability to adapt to environmental changes. This research examined the leaves of nine tree species, categorising them into three groups: MF50 (small trees: 50Y, 55Y, and 54Y), MF200 (old trees: 201Y, 208Y, and 210Y), and 350MF (the oldest trees: 356Y, 350Y, and 369Y). Figure 1 illustrates the morphological structure (A), stomatal density (B), and anatomical structure (C) of *P. euphratica* leaves at various ages. As trees aged, notable changes were observed in the leaf area, number of leaf veins, leaf thickness, and the area of the midrib vascular bundle in *P. euphratica* leaves.

**Figure 2:**
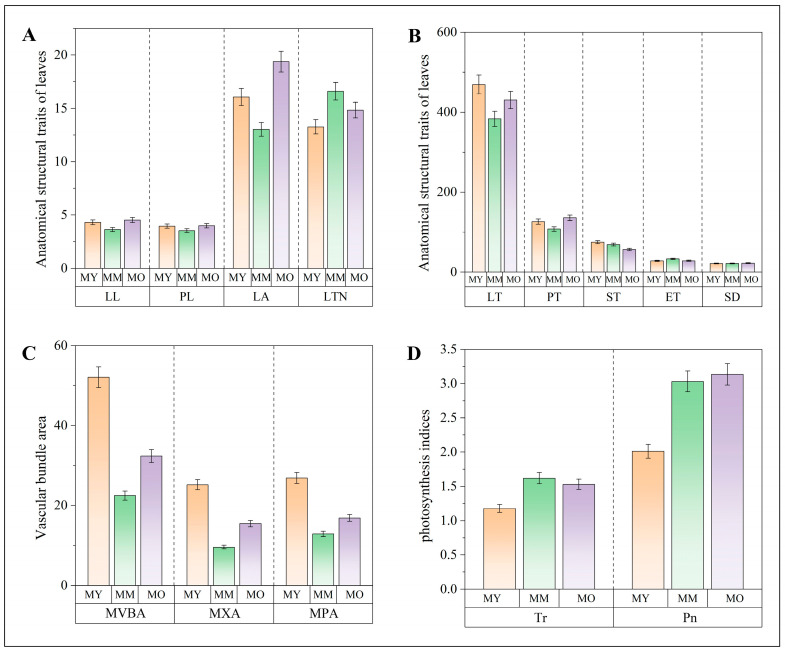
Comparison of leaf Morphological structure and Photosynthetic Physiological indicators of *P. euphratica* with Different Ages. (**A**) Leaf morphology indicators; (**B**) leaf anatomical structure indicators; (**C**) vascular bundle structure of main veins in leaves; and (**D**) leaf photosynthetic physiological indicators. LL: leaf length; PL: petiole length; LA: leaf area; LVN: leaf vein number; LT: leaf thickness; PT: palisade tissue; ST: spongy tissue; ET: epidermis thickness; SD: stomatal density; MVBA: main vascular bundle area; MXA: main vein xylem area; MPA: main vein phloem area; Tr: transpiration rate; and Pn: Net photosynthetic rate.

The analysis indicated that the average leaf area for trees aged 50, 200, and 350 years are 16.10, 13.03, and 19.38 cm^2^, respectively. Trees that are 200 years old have a smaller average leaf area than those that are 50 years old, while trees that are 350 years old have a larger average leaf area than the 50 and 200-year-old trees (Figure 2A). The quantity of leaf veins grew as the age of the trees ranged from 200 years to 350 years. This change could be attributed to the alteration in the leaf shape of *P. euphratica*, shifting from linear leaves without teeth to ovate leaves with teeth as the trees aged. The epidermal tissue structure of the broadest cross-section of *P. euphratica* leaves at various ages was examined. The findings indicated that, in comparison to those of smaller trees, the thickness of leaves and palisade tissue were reduced by 85.92 and 18.21 μm^2^, respectively, at the age of 200 years. A minor reduction was observed in the older trees (Figure 2B), while other factors like stomatal density (21.53, 21.69, and 22.5 mm^2^) did not exhibit any notable differences across the three age categories. At 200 years of age, the area of the main vascular bundle, xylem, and phloem decreased by 2.96, 1.56, and 1.40 cm^2^, respectively. By contrast, at 350 years of age, these areas showed slight increases of 0.99, 0.60, and 0.40 cm^2^, but these changes were not statistically significant in the last two age groups (Figure 2C). The findings from assessing the photosynthetic physiological indicators of *P. euphratica* leaves at various ages revealed that as the trees aged, the transpiration rate and net photosynthetic increased in middle-aged trees; however, no variation was observed in older trees (Figure 2D).

### 2.2. Anatomical Changes in Cambial Cells at Different Ages

**Figure 3:**
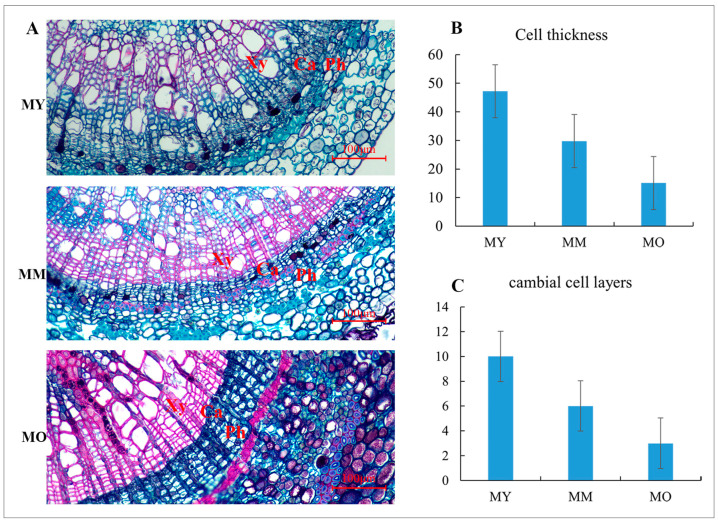
Anatomical structure of vascular cambium at different age and cell layers count. (**A**): MY (50 years), MM (200 years), MO (350 years); (**B**): Cell thickness; (**C**): cambial cell layers. Xy: xylem; Ph: Phloem; Ca: cambium.

The vascular cambium is a cell layer with an active division located between the secondary xylem and secondary phloem. The number of cell layers and the division activity within the vascular cambium significantly affect both wood yield and quality. To investigate whether the structure of the vascular cambium changes with age, we compared the anatomical structures of cambium bands in three different age groups of *P. euphratica* (Figure 3A). In the vascular cambium of 50-year-old *P. euphratica* branches, there are approximately 9–10 cell layers with dense cytoplasm, measuring a thickness of 47.2 μm (Figure 3B,C). In contrast, the vascular cambium of 200-year-old *P. euphratica* branches contains around 5–6 cell layers, with a thickness of 29.7 μm. The vascular cambium of 350-year-old branches has only 2–3 cell layers, with a thickness of 15.1 μm. The significant differences observed at these three stages suggest that the age of *P. euphratica* has a profound impact on the division and differentiation activities of its vascular cambium. These findings provide a cytological foundation for further studies on how the age of *P. euphratica* influences vascular cambium activity.

### 2.3. Transcriptome Analysis of Different Tree Ages Vascular Cambium of P. euphratica

Transcriptomes from trees aged 50, 200, and 350 years were constructed using de-novo transcript assemblies derived from paired-end Illumina RNA-seq reads to comprehensively study the gene expression patterns and identify key genes involved in primary and secondary growths in *P. euphratica*. Full-length transcript analysis was conducted using Illumina Novaseq 6000 sequencing. Following quality assessments and filtering, a total of 56.95 GB of clean data was produced, with average Q30 and GC percentages for each library recorded at 90.99% and 43.85%, respectively (Table S1). The comparison rate reflects the ratio of mapped reads to clean reads, providing a clear indication of the effectiveness of the transcriptome data utilization. The comparison statistics show that the alignment efficiency of reads with the reference genome for each sample ranged from 92.50% to 95.99%. The annotation of the reference genome is often imprecise, thus requiring adjustments to the original gene structure. When consecutive mapped reads extend beyond the original gene boundaries, the untranslated region of the gene is expanded both upstream and downstream to redefine the gene limits. This study successfully refined 3242 gene structures.

### 2.4. Functional Annotation

Functional annotation was conducted on 36,066 transcripts, with 35,664 (96.5%) of them successfully annotated across 8 protein databases. Specifically, 35,642 (96.5%) were annotated using the NR database, 29,219 (79.1%) with GO, 25,611 (69.3%) with KEGG, 16,982 (45.8%) with KOG, 14,060 (38.1%) with COG, 29,852 (80.8%) with Pfam, 26,227 (71%) with SwissProt, and 30,396 (82.3%) with eggNOG (Table S2). In the GO enrichment analysis, the most significantly represented terms were hydrolase activity (GO:0016787, 4092 transcripts, 14%) in the molecular function (MF) category, integral component of membrane (GO:0016021, 9086 transcripts, 31.1%) in the cellular component (CC) category, and cytokinin biosynthetic process (GO:0009691, 3464 transcripts, 11.8%) in the biological process (BP) category (Table S3). The KOG annotation results (Table S4) revealed that the largest number of unigenes was clustered into the functional category of “General function prediction only” (3651 transcripts, 21.5%), followed by “Posttranslational modification, protein turnover, chaperones” (2040 transcripts, 12%), and “Signal transduction mechanisms” (1759 transcripts, 10.3%). Approximately 233 (1.4%) transcripts were assigned to the cluster of “cell wall/membrane/envelope biogenesis”.

Based on the KEGG pathway analysis (Table S5), 136 pathways were identified, with most transcripts involved in the plant hormone signal transduction pathway (ko04075, 916 transcripts, 3.60%), the Plant–pathogen interaction pathway (ko04626, 916 transcripts, 3.60%), and the MAPK signaling pathway in plants (ko04016, 543 transcripts, 2.12%). Furthermore, 167 transcripts were associated with the phenylpropanoid biosynthesis pathway (ko00940), which is closely linked to lignin production.

### 2.5. Differentially Expressed Genes in P. euphratica at Different Ages

**Figure 4:**
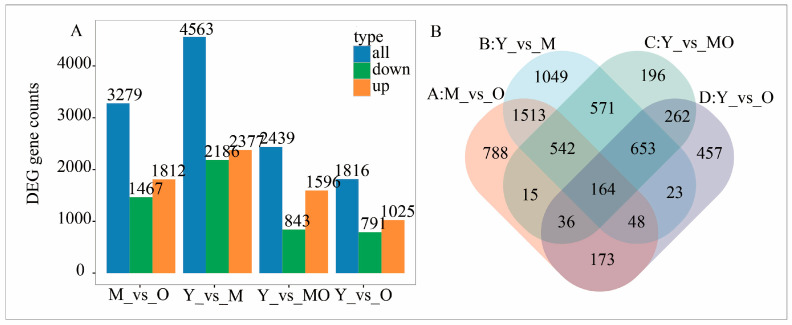
Differential gene expression analysis. Comparison of combined differentially expressed gene count (**A**) and Venn diagram of differential genes (**B**).

Four paired comparisons were performed to identify DEGs: MY versus MM, MY versus MO, MM versus MO, and MY versus MYO. A total of 6491 DEGs were identified (Figure 4). In the comparison of MY versus MM, 4563 DEGs were identified (2377 upregulated and 2186 downregulated). For MM versus MO, the number of DEGs reached 3279 (1812 upregulated and 1467 downregulated). In the MY versus MYO comparison, 2439 DEGs were identified (1596 upregulated and 843 downregulated), and the MY versus MO comparison revealed 1816 DEGs only (1025 upregulated and 791 downregulated).

**Figure 5:**
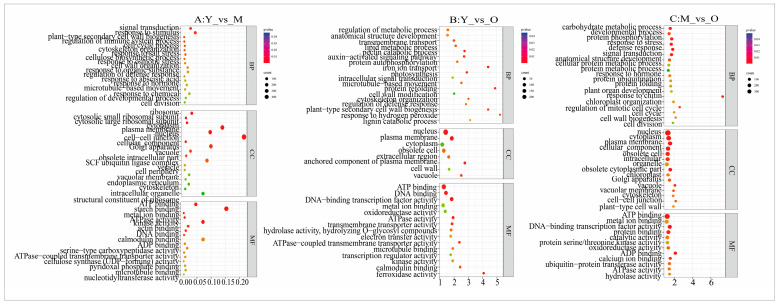
GO-enriched scatterplot of differentially expressed genes.

GO functional enrichment analysis was conducted for DEGs in each comparison group (Figure 5). The results identified 3627 DEGs enriched in 3974 GO terms in the MY versus MO comparison. Notably, the GO terms related to signal transduction (GO:0007165), xylan biosynthetic process (GO:0045492), and plant-type secondary cell wall biogenesis (GO:0009834) showed significant enrichment in the BP category. In the CC category, the GO terms for the ribosome (GO:0005840) and cytosolic small ribosomal subunit (GO:0022627) were significantly enriched. Additionally, the GO terms for structural constituents of ribosome (GO:0003735) and ATP binding (GO:0005524) exhibited significant enrichment in the MF category. A total of 1492 DEGs were enriched in 2100 GO terms in MY versus MO, and the GO terms of L-phenylalanine catabolic process (GO:0006559) and pectin catabolic process (GO:0045490) were significantly enriched in the BP category. The GO terms of plasma membrane (GO:0005886) and cell wall (GO:0005618) were significantly enriched in the CC category. The GO terms of pectate lyase activity (GO:0030570) and microtubule binding (GO:0008017) demonstrated significant enrichment in the MF category. A total of 2568 DEGs were enriched in 3295 GO terms in MM versus MO. The GO terms of L-response to chitin (GO:0010200) and defense response (GO:0006952) exhibited significant enrichment in the BP category. The GO terms of plasma membrane (GO:0005886) and nucleus (GO:0005634) were significantly enriched in the CC category. The GO terms of ADP binding (GO:0043531) and DNA-binding transcription factor activity (GO:0003700) were significantly enriched in the MF category.

KEGG pathway enrichment analysis was conducted for each comparative group’s DEGs (Figure S1). A total of 1689 DEGs were found to be enriched in 136 KEGG pathways in MY versus MM, 624 DEGs in MY versus MO, and 1155 DEGs in MM versus MO. Plant hormone signal transduction (ko04075), plant MAPK signalling pathway (ko04066), and phenylpropanoid biosynthesis (ko00940) were the three pathways that showed the greatest significance across the four comparison groups.

### 2.6. Expression Patterns of DEGs Related to Cell Division and Differentiation

**Figure 6:**
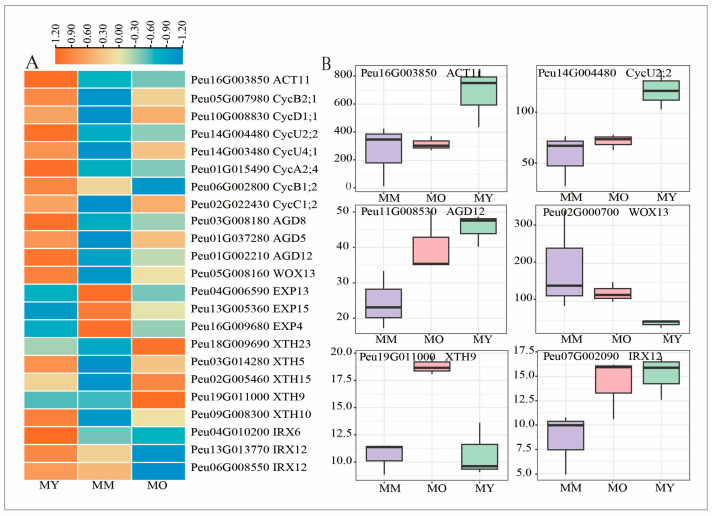
Expression profile of the DEGs involved in cell division and differentiation among three ages of *P. euphratica*. (**A**) Heatmap shows expression of DEGs associated with cell division and differentiation. (**B**) Boxplots show expression of all transcripts encoding *ACT11, CycU2;2, AGD12, WOX13, XTH9*, and *IRX12,* respectively.

The vascular cambium’s cells can produce new xylem and phloem by synchronised internal and external division and differentiation, which continuously promotes the thickening of roots and stems. Thus, the genes associated with cambium cell division and growth, including *WOX*, actin, cyclin, expansion, xyloglucan endotransglucosylase (*XTH*), and cellulose synthase genes, were explored. The majority of these DEGs exhibited noticeably reduced transcript levels in older trees. MY (50 years) had significantly higher transcript levels of cell division-related genes *ACT11* (*Peu16G003850)* (Figure 6A,B). Seven cyclin-related genes (Peu01G015490, Peu06G002800, *Peu05G007980*, *Peu10G008830*, *Peu14G004480*, Peu14G003480, and *Peu02G022430*) were significantly lower in MM (200 years) and MO (350 years) than in MY (50 years) (Figure 6A,B). The expression levels of the other two AGD transcripts (*Peu03G008180* and *Peu01G002210*) and *WOX13* (*Peu05G008160*) were higher in MY (Figure 6A,B).

The relative expression levels of two transcripts (*IRX6* and *IRX12*) encoding the catalytic subunit of cellulose synthase A were higher in MY than in other ages of *P. euphratica* (Figure 6A,B). This finding may be attributed to expansion, kinesin, and cyclin being crucial for plant growth and development because they promote cell division and differentiation. The expression levels of encoding expansions (*EXPA4, EXPA13*, and *EXPA15*) were higher in MM than in MY and MO. By contrast, five encoding xyloglucan endotransglucosylase transcripts (*Peu18G009690, Peu03G014280, Peu02G005460, Peu19G011000*, and *Peu09G008300*) were higher in MY and MO (Figure 6A,B). Notably, the expression of most genes associated with cell division did not differ significantly between MM and MO, but the expression of genes associated with cell expansion and differentiation was significantly different at the three ages.

The DNA methylation levels changed with tree age [26]. This study analyzed the differential expression of DNA methylation-related genes in the vascular cambium of trees at four different ages. The two transcripts of *MET1* and *RAD51* were upregulated in MO, whereas *DRM2* transcripts were downregulated in MO, *ROS1*, and *DME* and highly expressed in MM (Figure S2).

### 2.7. Expression Patterns of DEGs Related to Lignin_Biosynthesis

**Figure 7:**
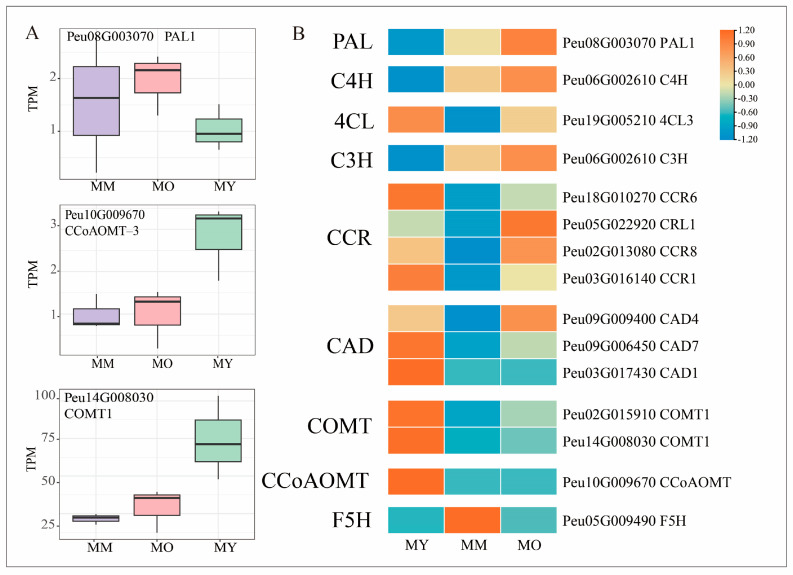
Expression profile of the DEGs involved in lignin biosynthesis among three ages of *P. euphratica*. (**A**) Heatmap shows expression of DEGs associated with cell division and differentiation. (**B**) Boxplots show expression of all transcripts encoding *PAL1*, *CCoAOMT-3* and *COMT1*, respectively.

One of the main components of plant cell walls is lignin, which is widely distributed in the xylem of vascular plants. Ten biosynthetic steps in the lignin monomer biosynthesis pathway were studied to identify important differential genes in lignin biosynthesis in the vascular tissues of Poplar at different ages. First, the hydroxylcinnamic acid produced by the phenylalanine pathway is the starting point for the specific lignin biosynthesis pathway. In the phenylpropanoid metabolic pathway, the transcripts of phenylalanine ammonia-lyase (*Peu08G003070*, *PAL*) and cinnamate-4-hydroxylase (*Peu06G002610*, *C4H*) were significantly upregulated in MM and MO (Figure 7), whereas the transcript of *Peu19G005210*, which encodes 4-coumarate-CoA ligase, was downregulated in MM.

The synthesis of lignin monomers requires several important enzymes (*C3H, COMT, CCoAOMT, F5H, CCR*, and *CAD*). *Peu06G002610* (*C3H*) was significantly downregulated in MY, upregulated in MM, and significantly upregulated in MO. Four CCR transcripts (*Peu18G010270, Peu05G022920, Peu02G013080*, and *Peu03G016140*) were downregulated in MM. The transcript encoding cinnamyl alcohol dehydrogenase (*CAD*), *Peu09G009400*, was downregulated in MM and upregulated in MO. By contrast, *Peu09G006450* (*CAD7*) and *Peu03G017430* (*CAD1*) were upregulated in MY and downregulated in MM and MO. Two transcripts encoding caffeic acid/5-hydroxyferulic acid O-methyltransferase (*Peu02G015910* and *Peu14G008030*, *COMT*) and one transcript encoding caffeoyl-CoA 3-O-methyltransferase (*Peu10G009670*, *CCoAOMT*) were significantly upregulated in MY and downregulated in MM and MO. Only one member of the phenolic acid 5-hydroxylase family (*Peu05G009490*, *F5H*) showed high expression in MM and low expression in MY and MO. In summary, the analysis of the most highly expressed members of each gene family revealed that, apart from caffeic acid/5-hydroxy conifer aldehyde O-methyltransferase, caffeic acid 3-O-methyltransferase, and ferulate 5-hydroxylase, the expression levels of monolignol pathway genes in the oldest tree were not lower than those in the youngest tree.

### 2.8. Expression Patterns of DEGs Related to Phytohormone

**Figure 8:**
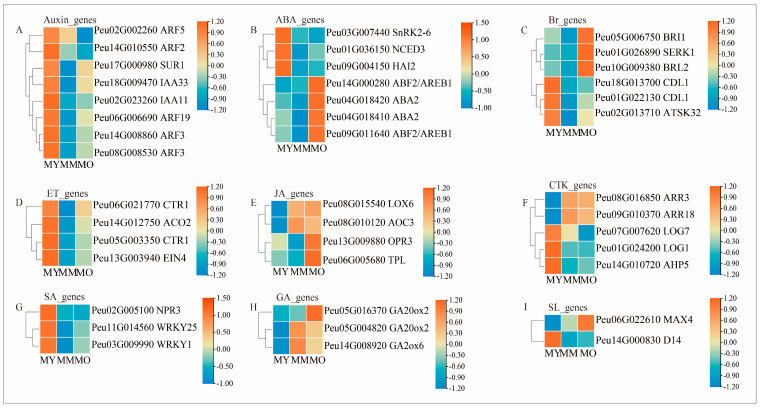
Expression profile of the DEGs involved in phytohormone among three ages of *P. euphratica*. (**A**): Auxin (Auxin); (**B**): Abscisic acid (ABA); (**C**): Brassinosteroids (BR); (**D**): Ethylene (ETH); (**E**): Jasmonic acid (JA); (**F**): Cytokinin (CTK); (**G**): Salicylic acid (SA); (**H**): Gibberellin (GA); (**I**): Strigolactone (SL).

Auxins, cytokinins, gibberellins, and other plant hormones are crucial signals for the development of plant vascular tissues. The coordinated interactions among these hormones can enhance the proliferation of the cambium and promote wood development. Auxin accumulates predominantly in the cambium and plays a vital role in maintaining the division and differentiation of cambial cells. Among the eight genes associated with auxin signal response, transduction, and induction (Figure 8A), the expression levels of auxin response family proteins involved in positive regulation (*Peu14G010550, Peu08G008530, Peu14G008860, Peu06G006690*, and *Peu02G002260*) were the highest in young trees (MY). The gene involved in negative regulation, *IAA11* (*Peu02G023260*), was upregulated in MY, with no significant differences observed in mature trees (MM) and older trees (MO). By contrast, the protein associated with auxin degradation (*Peu17G000980*) was upregulated in MY and MO, whereas its expression is downregulated in MM. Regarding the genes related to the ABA signaling pathway (Figure 8B), the transcription levels of *Peu14G000280, Peu09G011640, Peu04G018420*, and *Peu04G018410* were higher in older trees. Conversely, the transcription levels of *Peu01G036150* (*NCED3*), *Peu09G004150* (*HAI2*), and *Peu03G007440* (*SnRK2–6*) were upregulated in young trees. The two genes encoding the two-component response regulator *ARR3* (*Peu08G016850* and *Peu09G010370*) exhibited higher relative expression in MM and MO. Meanwhile, the genes encoding *APH5* (*Peu14G010720*), *LOG1* (*Peu01G024200*), and *LOG7* (*Peu07G007620*) were highly expressed in MY (Figure 8F).

Inhibiting the biosynthesis of brassinosteroids (BRs) can reduce secondary lignification while conversely promoting secondary growth and wood formation in Poplar. Amongst the six BR-related genes (Figure 8C), the transcripts of *CDL1* and *ATSK32* were upregulated in MY, and *Peu05G006750* (*BRI1*), *Peu10G009380* (*BRL2*), and *Peu01G026890* (*SERK1*) demonstrated relatively high expression levels in MO. Gibberellin is a key signaling molecule for plant growth, similar to auxin. Among the three genes related to gibberellin biosynthesis and metabolism (Figure 8H), the expression levels of *Peu05G004820* (*GA20ox2*) and *Peu14G0008920* (*GA2ox6*) were high in MM and MO. By contrast, the *GA20ox2* transcript *Peu05G16370* was upregulated in MO, with no significant difference between MM and MO. Four genes related to the ethylene signaling pathway (*Peu06G021770*, *Peu055G003350*, *Peu14G0012750*, and *Peu13G003940*) were upregulated in MY (Figure 8D). In the jasmonic acid signaling pathway, the transcripts encoding *LOX6* (*Peu08G015540*) and *AOC3* (*Peu083010120*) were upregulated in MM and MO. The transcripts encoding *OPR3* and TPL (*Peu13G0009880* and *Peu06G005680*, respectively) were highly expressed in MY (Figure 8E).

### 2.9. Expression Patterns of TF-Related DEGs

**Figure 9:**
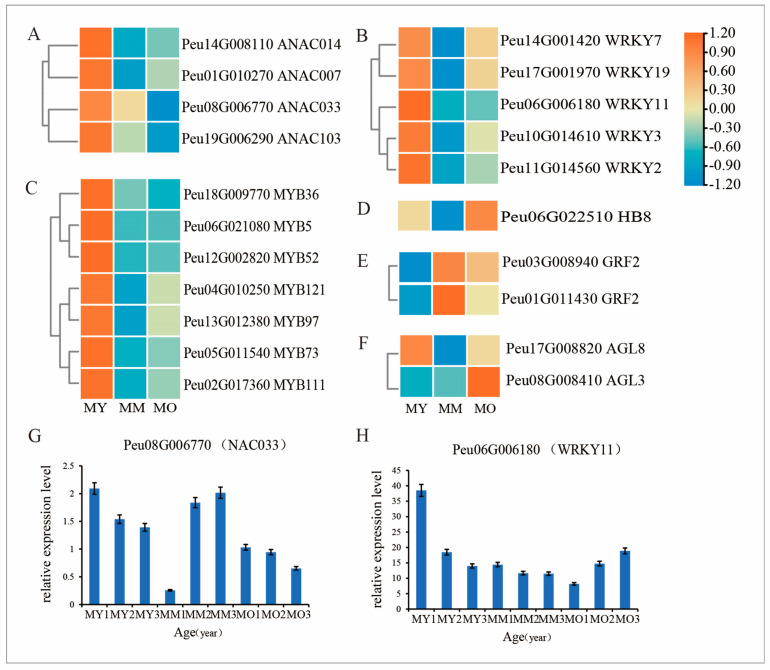
Expression profile of the DEGs involved in transcription factors (TF) among three ages of *P. euphratica*; (**A**): NAC TF; (**B**): WRKY TF; (**C**): MYB TF; (**D**): HB TF; (**E**): GRF TF; (**F**): AGL TF; Transcript levels of (**G**) *NAC033* and (**H**) *WRKY11* among more ages.

The activity of the vascular cambium is highly regulated and controlled by precise molecular mechanisms. In addition to plant hormones and signaling peptides, transcription factors play a crucial role in modulating the functions of the vascular cambium. This study identified seven *MYB*, five *WRKY*, four *NAC*, two *GRF*, two *MADS-box*, and one *HD-ZIP* differential genes. The findings indicate that a significant number of transcription factors are involved in regulating the activity of the vascular cambium in *P. euphratica*, with their expression levels varying according to tree age (Figure 9). Among them, the NAC transcripts *Peu02G013660* (*ANAC014*) and *Peu01G010270* (*ANAC007*) were upregulated in MY but showed no differential expression in MM and MO. Conversely, *Peu08G006770* (*ANAC033*) and *Peu19G006290* (*ANAC103*) were upregulated in MY and downregulated in MO, exhibiting significant differences among the three groups (Figure 9A, G). Within the *WRKY* family, the expression levels of *WRKY7, WRKY19, WRKY11, WRKY3*, and *WRKY2* transcripts were upregulated in MY (Figure 9B, H).

Additionally, differences were observed in the expression of *MYB*, a key regulatory factor associated with vascular differentiation and phenylpropanoid metabolism in plants. Seven MYB transcription factors (*MYB5, MYB36, MYB52, MYB73, MYB97, MYB111*, and *MYB121*) were upregulated in MY but downregulated in MM and MO (Figure 9C). The third homologous domain related to meristematic tissue function, the leucine zipper protein (*HD Zip III*), plays a crucial role in the activity of the plant vascular cambium. In MO, *Peu06G022510*, which encodes *HB8*, was highly expressed (Figure 9D). Furthermore, GRF and MADS-box transcription factor genes are recognised for their roles in regulating meristem activity, floral organ identity specification, and organ development. The two DEGs encoding GRF (*Peu03G008940* and *Peu01G011430*) were highly expressed in MM and MO (Figure 9E). Between the two DEGs encoding MADS (Figure 9F), *Peu17G008820* (*AGL8*) was upregulated in MY, whereas *Peu08G008410* (*AGL3*) was highly expressed in MO.

### 2.10. Validation of Candidate Differential Genes in Vascular Cambium

**Figure 10:**
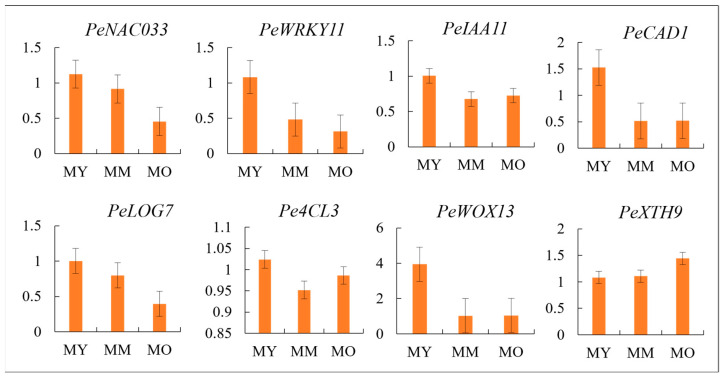
qRT-PCR data for eight DEGs expression patterns.

To validate the accuracy of the transcriptome data, we selected the genes *WOX13* (*Peu05G008160.1*), *XTH9* (*Peu19G011000.1*), *NAC033* (*Peu08G006770.1*), *WRKY11* (*Peu06G006180.1*), *IAA11* (*Peu02G023260.1*), *LOG7* (*Peu07G007620.1*), *CAD1* (*Peu03G017430.1*), and *4CL3* (*Peu19G005210.1*) for real-time quantitative reverse transcription polymerase chain reaction (RT-qPCR). These genes are associated with vascular cambium activity in urochordates of different ages. The relative expression profiles of the eight candidate genes obtained by RT-qPCR were consistent with the expression trends observed in the transcriptome data (Figure 10). This confirms the high reliability of the transcriptome data and their accuracy in reflecting the gene expression levels in this study.

## 3. Discussion

Evolutionary biologists define aging as a decline in survival rates and reproductive capabilities associated with age, signifying a deterioration of various physiological functions in individuals considered elderly [27]. The aging process in plants is influenced by a range of factors, including biological elements such as competitive relationships among plants and their genetic makeup [28], and abiotic factors like drought, salinity, extreme temperatures, and heavy metal stress resulting from adverse environmental conditions [29,30]. Among the various traits of plants, leaf characteristics most effectively reflect their adaptability to environmental changes [31]. Leaf structure undergoes changes with age and represents a complex and highly organised developmental process that precisely regulates the timing and spatial initiation of leaf aging [32,33].

With changes in the environment or stages of individual development, leaves often display variations in shape, thickness, and internal anatomical structure [34]. The morphology and anatomical characteristics of *P. euphratica* leaves at various ages were studied, and the findings revealed that older trees possess a larger leaf area than younger trees [1,35,36]. These results indicate that the allometric growth of leaf area in relation to tree age reflects the relationship between individual developmental stages and resource allocation. This finding may represent an active coping strategy that leaves have evolved in response to external stimuli, as well as growth and development processes [37]. Previous studies suggest that thicker leaves and a more robust epidermal system are associated with larger epidermal cells, which enhance the leaves’ capacity for water storage, retention, and drought resistance [38,39]. In this study, leaf thickness exhibited a trend of initially decreasing and then increasing with tree age, whereas no significant difference in leaf epidermal cell thickness was observed between age classes. An increase in leaf thickness can reduce internal water loss in plants and enhance their water retention capacity, whereas a decrease in leaf thickness can improve the photosynthetic efficiency of plants in low-light conditions [40,41]. The thicker the palisade tissue, the denser the leaf structure, indicating a tighter arrangement of mesophyll cells, a higher photosynthetic rate, and increased drought resistance in the plant [1]. In this study, the thickness of palisade tissue was greater in older trees than in younger ones, whereas the variation pattern of sponge tissue thickness exhibited an opposite trend. The results indicated that the mature *P. euphratica* trees still possessed well-developed palisade tissue, enabling them to withstand extreme drought conditions.

Stomata are crucial structures in plant leaves that regulate the exchange of gases between plants and their external environment. They also serve as the primary pathway for water loss in plants. Stomata play a vital role in optimising the processes of photosynthesis and transpiration [35]. The well-developed vascular bundles in the main veins of leaves demonstrate the plant’s strong transport capacity, enhancing its drought resistance [42,43]. Studies have found a trade-off between stomatal density and leaf vein density in the leaves of woody plants. This relationship exhibits significant synergistic variation, reflecting the alignment of stomatal transpiration with water demand and the leaf vein system with water supply [44,45]. In this study, no change in stomatal density was observed between the MY and MO age classes, whereas the number of leaf veins exhibited a pattern of initial increase followed by a decrease. However, the areas of the main vein vascular bundle, main vein xylem, and main vein phloem decreased as the tree aged. The results indicated that the growth status of *P. euphratica* diminished to a certain extent with increasing tree age. However, the consistent stomatal density enabled older *P. euphratica* to maintain a balance amongst physiological activity, water supply, and leaf water loss, in conjunction with leaf vein density, in arid environments.

At various stages of the life cycle, as age increases, the growth rate of plants decreases, stress resistance diminishes, photosynthesis weakens, and a series of antioxidant enzymes within the cells undergo dynamic changes [46]. However, this study found that as tree age increased, the net photosynthetic rate and transpiration rate exhibited an upward trend. This observation aligns with previously reported significant increases in the net photosynthetic rate, transpiration rate, and stomatal conductance of *P. euphratica* from young trees (A) to old trees (C) [47]. In arid environments, desert plants activate their internal defence mechanisms, modify their morphological structures to enhance photosynthesis, and accumulate greater amounts of dry matter along with various stress-resistant physiological and biochemical substances to meet their growth needs under adverse conditions. In summary, the leaf traits of *P. euphratica* indicate that older individuals exhibit greater resistance to external disturbances and possess superior resource utilization capabilities than their younger counterparts. As the age of the tree increases, *P. euphratica* individuals can enhance photosynthesis, reduce transpiration, and accelerate growth by adjusting their leaf morphology, nutrient characteristics, and chlorophyll content. This adaptation allows them to store energy in preparation for the breeding season and to cope with environmental stressors. Overall, the findings regarding these morphological and anatomical structures, along with photosynthetic physiological indicators, demonstrate that ancient *P. euphratica* trees remain in a healthy adult state, maintaining their “youthful vitality”, and have not yet entered the aging phase.

The vascular cambium can divide and differentiate, producing secondary phloem and secondary xylem. While previous studies have investigated the vascular cambium, research on its activity across different tree ages remains limited. Rossi et al. found that in many species, the vascular cambium of older trees becomes active later and remains active for a shorter duration compared to that of mature trees [48]. To better understand the genes related to vascular cambium activity and their regulatory networks in *P. euphratica* at different ages, this study conducted anatomical observations on the vascular cambium of *P. euphratica* branches from trees of three different ages. The results showed that, compared to *P. euphratica* trees at 200 and 350 years old, the vascular cambium of trees at 50 years old had more cell layers, with up to 9–10 layers, while that of trees at 350 years old only had 2–3 layers. This finding is consistent with Zhao et al.’s study of young and ancient Ginkgo trees [49], which showed that the vascular cambium of young Ginkgo trees has significantly more cell layers than that of ancient trees. These results suggest that the number of cell layers in the vascular cambium gradually decreases with increasing tree age, and that the number and division capacity of cells in the vascular cambium are closely associated with secondary growth in woody plants.

Cell division and differentiation are crucial processes for maintaining the functionality of the cambium. The capacity of vascular stem cells to generate secondary vascular tissue means that the ability of these cambium cells to divide and differentiate directly influences their growth rate and overall yield [50]. Research has shown that the overexpression of *PttPXY* and *PttCLE41* (which are homologous to *A. thaliana TDR/PXY* and *CLE41*, respectively) in *Populus tomentosa* is associated with slow plant growth [51]. *PtrHB7* (*HD Zip III*) is significantly enriched in the vascular cambium and can regulate the differentiation of cambium cells by either upregulating or downregulating *PtrHB7* [52]. The formation of cell walls and secondary walls has been shown to be associated with gene expression in the cambium, including *XTH*, pectin methylesterase, beta-1,3-glucanase, and endo-1,4-beta-glucanase, all of which are present in the vascular cambium [53]. In this study, a significant number of genes were associated with cell division amplification, such as *ACT11*, cyclins, *AGD*, *WOX13*, and encoding cellulose synthase A catalytic subunits (*IRX6* and *IRX12*), all of which were highly expressed in young trees (MY). By contrast, transcripts encoding xyloglucan endoglucanase were predominantly expressed in older trees. In summary, these findings suggest that the division and expansion capabilities of the vascular cambium cells in *P. euphratica* are diminished in ancient trees; however, their ability to form secondary xylem is enhanced.

Lignin is a primary component of plant cell walls, predominantly located in the xylem of vascular plants. It provides essential mechanical support [54,55,56] and plays a crucial role in maintaining normal plant growth and development [57,58,59,60]. In this study, the expression levels of nine genes involved in lignin biosynthesis exhibited no consistent pattern across three age classes; however, they were highly expressed in both young and old trees. These results suggest that lignin biosynthesis is not constrained by the age of the trees.

Plant hormones play a crucial role in plant growth and development by regulating cambium activity, cell expansion, and leaf senescence [61,62]. However, the research on the effects of plant hormones on the vascular cambium across different tree ages is relatively few. Current research showed that ABA increases during the transition from juvenile to adult stages in several plant species; however, the changes in ABA levels in adult and ancient trees remain unexplored [63]. Auxin is regarded as a crucial signal that regulates the proliferation of vascular cambium cells and determines the identity of the vascular cambium meristem in perennial woody plants [64]. In the study of *Pinus taeda*, researchers discovered that the auxin-inducible gene encoding 5NG4 is regulated differently in adult and juvenile plants [65]. The present study identified numerous hormone-related genes in the cambium of *P. euphratica*, including auxin, cytokinin, ethylene, and abscisic acid. Notably, compared with 50-year-old *P. euphratica* trees, the cambium of older trees showed down-regulated genes associated with auxin, ethylene, salicylic acid, and certain cytokinins including auxin response factors such as *ARF* and *LOG7*. Conversely, genes related to JA, GA, and some ABA and Br hormones were upregulated in ancient trees. Examples include the ABA response element binding protein (*ABF*/*AREB1*), somatic embryogenesis-related receptor protein kinase (*SERK1*), the co-repressor protein (*TPL*) in jasmonic acid signaling, and the key enzyme for gibberellin biosynthesis (*GA20ox2*). These findings suggest that as trees age, the growth-promoting hormone genes decrease, whereas the growth-inhibiting hormone genes tend to increase.

Transcription factors play a crucial regulatory role in the development of plant vascular cambium. In a study comparing juvenile *Cunninghamia lanceolata* (3 years old) with mature *C. lanceolata* (35 years old), the expression of *HD-Zip III* in juvenile *C. lanceolata* was significantly lower than in adult specimens. The expression levels of genes encoding HD-Zip III transcription factors in juvenile *C. lanceolata* were lower than those in adult *C. lanceolata* [13]. Similar findings were observed in this study, where the *HB8* gene (*Peu06G022510*), which encodes members of the *HD-Zip III* family, was expressed at lower levels in MY and MM. This result further supports the idea that *HD-Zip III* plays a role in regulating vascular cambium activity. In addition to HD-Zip III, this study identified several other families of transcription factors involved in the regulation of vascular cambium activity during juvenile and adult stages, including *WRKY*, *NAC*, *MADS-box*, *MYB*, and *GRF*. This finding indicates that a significant number of transcription factors are involved in regulating vascular cambium activity in *P. euphratica*, with their expression levels varying according to tree age. For instance, transcription factors WRKY and NAC, which are closely associated with plant growth, development, abiotic stress, and aging, exhibited differential expression in the vascular cambium of *P. euphratica* at various ages. Five genes encoding WRKY family transcription factors (*Peu14G0001420, Peu17G001970, Peu06G006180, Peu10G014610*, and *Peu11G014560*) and four genes encoding NAC family transcription factors (*Peu14G0008110, Peu01G010270, Peu08G006770*, and *Peu19G006290*) were expressed at higher levels in the vascular cambium of *P. euphratica* at 50 years of age than in those at 200 and 350 years of age. This finding aligns with previous studies on radiation pine and white spruce [66]. Additionally, differences were noted in the expression of the key regulatory factor MYB, which is related to vascular differentiation and phenylpropanoid metabolism in plants. The seven genes encoding MYB (*MYB5, MYB36, MYB52, MYB73, MYB97, MYB111*, and *MYB121*) exhibited higher expression levels in the vascular cambium of *P. euphratica* at the young (MY) age than at the mature (MO) age. By contrast, the expression levels of growth regulators GRF (*GRF2* and *GRF3*) and MADS-box (*AGL3* and *AGL8*), which are involved in regulating meristematic activity, were significantly higher in older trees. These research findings suggest that the transcription factors in the vascular cambium during the growth and development of *P. euphratica* respond to changes in tree age.

## 4. Conclusions

This article focuses on *P. euphratica*, the oldest extant angiosperm in the Salicaceae family, as the research system. Using high-throughput sequencing technology combined with experimental techniques such as cytology, molecular biology, and bioinformatics, the study investigates the vascular cambium of *P. euphratica*. In the anatomical analysis of the vascular cambium, it was observed that as the age of *P. euphratica* increases, both the number and thickness of cambium cells decrease. In the differential gene analysis, genes related to cell division and differentiation, hormone-related genes, lignin biosynthesis genes, and aging-related transcription factors exhibited upward or downward trends with the increase in tree age, suggesting that these genes, whose expression patterns vary with tree age, play an important regulatory role in the aging process of *P. euphratica*. In summary, the decrease in the number of cambium cells and the expression levels of genes associated with cell division, differentiation, and expansion across different ages of *P. euphratica* indicates a significant decline in cambium activity in ancient trees, leading to a reduction in growth rate. Furthermore, the expression patterns of genes related to auxin and cytokinin in the cambium of *P. euphratica* at different ages were found to be opposite to those of genes related to abscisic acid, indicating that these plant hormones respond to changes in tree age. This study provides a scientific theoretical basis for understanding the lifespan of ancient *P. euphratica* trees by exploring the transcriptional regulation of the vascular cambium at different tree ages.

## 5. Materials and Methods

### 5.1. General Situation

The experimental materials were collected from the *P. euphratica* forest reserve in Minfeng County, Xinjiang Uygur Autonomous Region (longitude 82°47′38.5332″ E, latitude 37°44′6.3312″ N). In late June 2022, we conducted a preliminary age estimation of the *P. euphratica* by measuring the diameter at breast height (DBH) of individual *P. euphratica* trees and applying the age estimation formula (estimated age = DBH ÷ 2 ÷ average annual growth). Based on this, nine poplar trees of different ages were selected as experimental materials (Table S1). The climate of the study area is characterized by hot and dry conditions, with an average annual rainfall of about 31 mm, an average annual temperature of 10.8 °C, annual evapotranspiration exceeding 2750 mm, and an average annual sunshine duration of 2842 h. The region is a typical temperate continental arid desert climate zone.

### 5.2. Biological Material

The experimental materials for this study were categorized into three age groups: 50 years (Young Trees in Minfeng County MY), 200 years (Middle-aged Trees in Minfeng County MM), and 350 years (Old Trees in Minfeng County MO) (Figure S3). In June 2022, three uniformly growing *P. euphratica* trees were selected from each age group within the designated experimental area as test materials. The bark was removed using a sharp chisel at a height of 1.3 m from the base of the tree, and the cambium and phloem beneath the bark were scraped away with a small knife. Simultaneously, a sample block of xylem measuring 3.0 cm × 3.0 cm × (1.0–2.0) cm (length × width × depth) was chiseled from the same location and immediately placed into an RNAse-free Eppendorf centrifuge tube. After rapid freezing with liquid nitrogen, the samples were stored at −80 °C for future use.

### 5.3. Anatomical Observations of the Vascular Cambium

Due to difficulties in obtaining materials from the *P. euphratica* Nature Reserve in Minfeng County, Xinjiang, anatomical samples for this experiment were instead obtained from current-year branches of the same tree crown orientation as the previously mentioned samples. Using a dissecting knife, we cut a stem segment from the third node, where the leaves are located, on the current-year branches. The samples were then immediately fixed in a 70% FAA solution for at least 24 h. Subsequently, the material was dehydrated, wax-infiltrated, and embedded using the paraffin sectioning method. Thin sections (10 µm) were cut, stained with 1% safranin and 0.5% fast green, and examined under a microscope to observe tissue changes. The patterns of these changes were documented through microscopic images.

### 5.4. Morphological and Structural Analysis

(1) Measurement of Leaf Morphology and Structure: Leaf morphology and structural traits of *P. euphratica* were measured using a scanner (MRS-9600TFU2) and ImageJ (v 1.44) software. Parameters recorded included the number of leaf veins (LTN), leaf area (LA), leaf length (LL), and petiole length (PL). (2) Determination of Leaf Stomatal Indexes: Healthy, undamaged leaves were selected, and a thin, even layer of nail polish was applied to the abaxial (lower) surface of the leaves. After the nail polish had completely dried, transparent adhesive tape was carefully applied to the dried area, ensuring the tape lay flat and fully covered the target region. The tape was gently pressed to adhere completely to the nail polish layer. The tape was then carefully removed from the leaf surface and mounted onto a clean microscope slide, minimizing the presence of air bubbles. The stomatal density was observed and recorded under a light microscope. (3) Determination of Leaf Tissue Anatomical and Structural Indexes: Cross-sections were prepared from the widest part of the *P. euphratica* leaf and immediately fixed in a 70% FAA solution for preservation. Sections were prepared using the paraffin embedding method, cut to a thickness of 8 µm, double-stained with safranin and fast green, and sealed with neutral resin. The following anatomical and structural parameters were measured under a Leica microscope: leaf thickness (LT), main vein xylem area (MXA), palisade tissue thickness (PT), main vein phloem area (MPA), main vascular bundle area (MVBA), epidermal thickness (ET), and spongy tissue thickness (ST). Leaf blades were taken from current-year branches in the same orientation of the canopy of the nine samples.

### 5.5. Illumina RNA-Seq Library Construction and Sequencing

Total RNA was isolated from nine vascular cambium samples (three tree ages, three replicates per tree age) using the RNAprep Pure Plant Kit (Tiangen, Beijing, China) according to the manufacturer’s instructions. RNA concentration and purity were measured using a NanoDrop 2000 (Thermo Fisher Scientific, Wilmington, DE). RNA integrity was assessed using the RNA Nano 6000 Assay Kit with the Agilent Bioanalyzer 2100 system (Agilent Technologies, Santa Clara, CA, USA).

A total of 1 μg RNA per sample was used as input material for RNA sample preparations. Sequencing libraries were generated using the Hieff NGS Ultima Dual-mode mRNA Library Prep Kit for Illumina (Yeasen Biotechnology (Shanghai) Co., Ltd., Shanghai, China) following the manufacturer’s recommendations, and index codes were added to attribute sequences to each sample. Then, 3 μL USER Enzyme (NEB, Ipswich, MA, USA) was used with size-selected, adaptor-ligated cDNA at 37 °C for 15 min followed by 5 min at 95 °C before PCR. PCR was performed with Phusion High-Fidelity DNA polymerase, Universal PCR primers, and Index (X) Primer. Finally, PCR products were purified (AMPure XP system), and library quality was assessed on the Agilent Bioanalyzer 2100 system. The libraries were sequenced on an Illumina NovaSeq platform to generate 150 bp paired-end reads, according to the manufacturer’s instructions. The raw reads were further processed using the bioinformatics pipeline tool BMKCloud (www.biocloud.net) online platform.

### 5.6. Bioinformatic Analysis of Illumina Data

To obtain high-quality, clean reads, the raw data were further filtered using FASTQ. Bemac employs HISAT2 (v2.2.1) [67] software to align the clean reads efficiently and accurately with the *P. euphratica* reference genome, determining the positioning of the reads on the reference genome. The reads were then assembled using StringTie (v2.2.3) [68] to reconstruct the transcriptome for further analysis. The StringTie Reference Annotation-Based Transcript (RABT) assembly method was used to construct and identify both known and novel transcripts from the HISAT2 alignment results.

Gene function was annotated based on the following databases: Nr (NCBI non-redundant protein sequences), Pfam (Protein family), KOG/COG (Clusters of Orthologous Groups of proteins), Swiss-Prot (a manually annotated and reviewed protein sequence database), KO (KEGG Ortholog database), and GO (Gene Ontology). Gene Ontology (GO) enrichment analysis of the differentially expressed genes (DEGs) was implemented using the clusterProfiler package, based on Wallenius non-central hypergeometric distribution, which adjusts for gene length bias in DEGs. KEGG pathway enrichment of DEGs was performed using the KOBAS database and clusterProfiler (v4.14.4) software.

### 5.7. Differential Expression Analysis

We used StringTie, normalized by the maximum flow algorithm using TPM 2.0 [68] (Fragments Per Kilobase of transcript per Million fragments mapped), as a measure of transcript or gene expression level. Differential expression analysis was conducted using the EBSeq R package based on the gene count values for each sample. Significant differential expression was determined with a Fold Change ≥ 1.5 and *p*-value < 0.05 as thresholds. The differentially expressed genes (DEGs) were subjected to enrichment analysis of GO and KEGG pathways.

### 5.8. RT-qPCR Validation

To further validate the accuracy of the sequencing data, we selected eight differentially expressed genes (DEGs) with significantly varied expression across different tree ages for qRT-PCR validation. Candidate gene-specific primers for quantitative real-time PCR (qRT-PCR) were designed using the NCBI Primer-BLAST tool (https://www.ncbi.nlm.nih.gov/tools/primer-blast/index.cgi (accessed on 26 November 2024)) (Table S7). Reverse transcription was performed using the PrimeScript™ II 1st Strand cDNA Synthesis Kit (Code No. 6210A). qRT-PCR was conducted with the 2X ChamQ Blue Universal SYBR qPCR Master Mix, and amplification was performed on the Applied Biosystems 7500 Real-Time PCR System. The expression levels of the candidate genes were normalized to the poplar housekeeping gene Actin as an internal reference. The relative expression levels of these candidate genes were calculated using the 2^−ΔΔCt^ method.

## Data Availability

Data are contained within the article and Supplementary Materials.

## Supplementary Materials

The following supporting information can be downloaded at: https://www.mdpi.com/article/10.3390/plants13243549/s1, Figure S1: KEGG enrichment bubble plot of differentially expressed genes; Figure S2: Expression patterns of DEGs related to DNA methylation-related genes; Figure S3. Samples of Poplar of different ages. MY: 50years, MM: 250years, MM: 350years; Table S1: Details information of the tree samples; Table S2: Sequencing Data Quality Control Statistics; Table S3: Statistical Table of Gene Function Annotations; Table S4: Gene functional enrichment analysis; Table S5: KOG annotation analysis; Table S6: KEGG enrichment pathway; Table S7: Details of the primers for the 8 candidate genes.
