# Supplementary material for: Multi Characteristic Analysis of Vascular Cambium Cells in Populus euphratica Reveals Its Anti-Aging Strategy
Source: Plants (Basel). 2024 Dec 19;13(24):3549. doi: 10.3390/plants13243549 (PMC11677677; doi:10.3390/plants13243549)
Supplement: Supplementary file 1 [file plants-13-03549-s001.zip › Supplementary Materials.pdf]

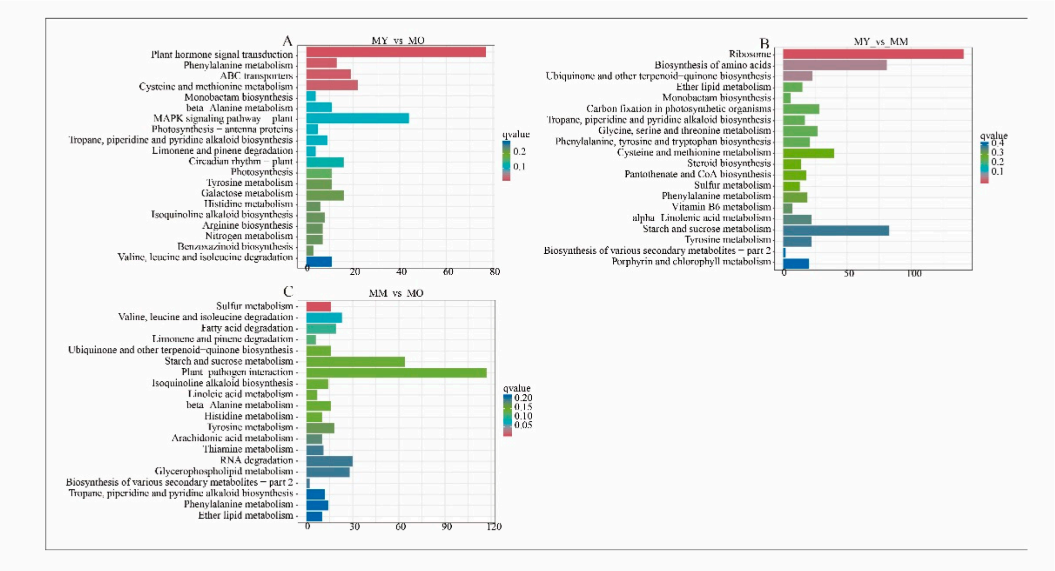

Figure S1. KEGG enrichment bubble plot of differentially expressed genes.

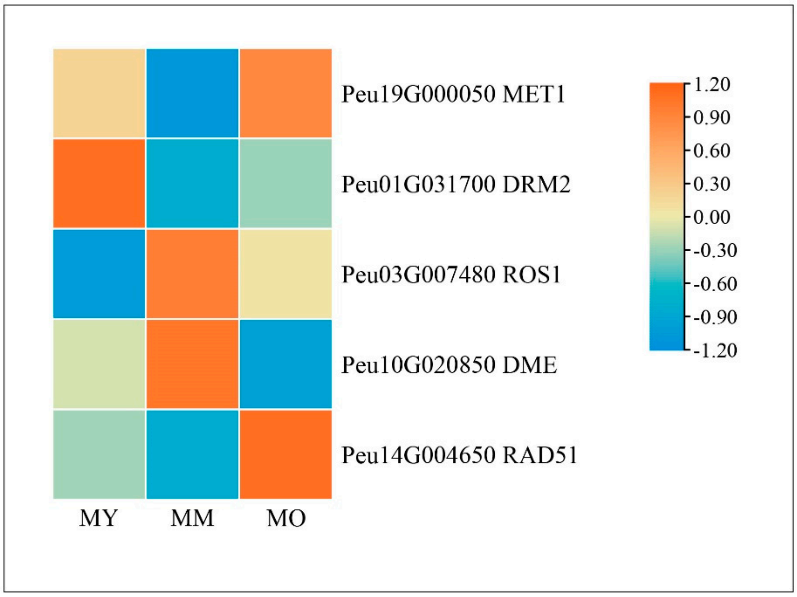

Figure S2. Expression patterns of DEGs related to DNA methylation-related genes.

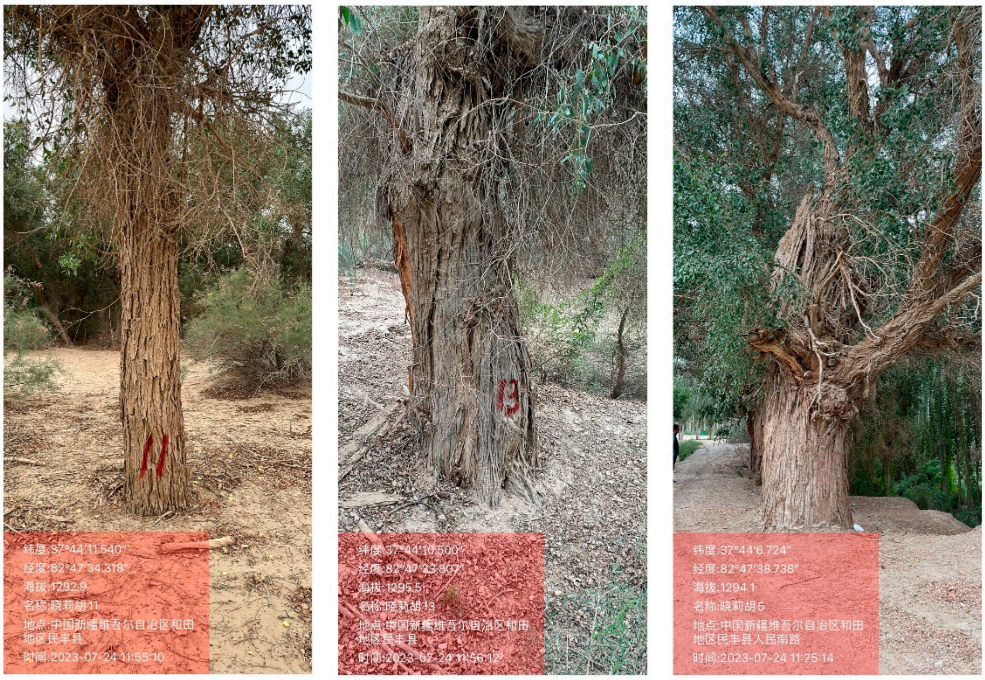

MY(50 years) MM(250 years) MO(300 years)

Figure S3. Samples of poplar of different ages. MY: 50years, MM: 250years, MM: 350years.
